# Maximizing coverage, reducing time: a usability evaluation method for web-based library systems

**DOI:** 10.1038/s41598-022-11215-7

**Published:** 2022-05-04

**Authors:** Shumaila Iqbal, Naveed Ikram, Salma Imtiaz, Saima Imtiaz

**Affiliations:** 1 Faculty of Computing, Riphah International University, Islamabad, Pakistan; 2 Department of Computer Science and Software Engineering, International Islamic University, Islamabad, Pakistan

**Keywords:** Computer science, Software

## Abstract

The usability of a Web Based Library System (WBLS) is an important quality attribute that must be met in order for the intended users to be satisfied. These usability quality attributes are available in two forms: general to web systems and domain-specific. It must be evaluated through some evaluation method such as checklist. Many evaluation checklists have been proposed, although they mostly facilitate the evaluation of WBLS’s general usability aspects, but they lack in covering domain-specific usability aspects of WBLS. There is a need to define domain specific usability aspects to maximize the usability for such systems. The purpose of this research is to develop and validates a usability evaluation checklist that supports the evaluation of general as well as specific usability aspects of WBLS. To accomplish this, a control experiment was conducted in the first phase with undergraduate students to develop a usability evaluation checklist that includes both general and specific usability aspects. Another controlled experiment will be used in the second phase to evaluate the effectiveness and efficiency of the proposed checklist with the existing checklist as "Academic Library Website Evaluation Checklist". The manual and statistical result shows that, the proposed usability evaluation checklist is effective with maximum coverage of general and specific usability aspects. Furthermore, the proposed checklist is equally efficient while identifying the usability errors in WBLS. The proposed checklist is beneficial for the academia as well as industry to evaluate the usability of WBLS to an optimal level.

## Introduction

Usability is a critical success factor of a Web Based Library System (WBLS). It is a quality attribute to assess the ease of the user interface with multi-dimensional attributes of usability being learn-ability, efficiency, memorability, low error rate and satisfaction^1,2^. There exists different approaches to evaluate usability^2^ such as heuristic evaluation^3^, cognitive walk through^4^, formal usability inspections^5^, pluralistic walk through^6^ and checklists^7^. The metrics used to measure usability are diverse such as task completion time, error rates, subjective satisfaction, perceived workload, assessment of a work product’s quality, feeling of enjoyment and ease-of-use etc.^8,9^. In this research, the checklist-based usability evaluation method is adopted and measured. The usability in terms of effectiveness correspond to number of errors and efficiency i.e., time taken to complete the identify the usability errors in WBLS. WBLS and related technologies such as Next Generation Catalogue (NGC) and Online Public Access Catalogue (OPAC) have become widely used in the modern era. This phenomenon is becoming increasingly popular as a means of removing difficulties in using WBLS and enhancing rapid and easy information retrieval^10–12^. The quality of functionality delivered to library users, namely students and researchers, is a crucial aspect impacting library performance in WBLS. The simple and interactive user interface serves as a link between the WBLS and the end users. A major difficulty in creating WBLS is to provide an interface that is attractive in terms of its general functionality, like that of all other web-based systems, as well as the domain-specific features of library systems^13^. To assess the quality in terms of usability, the checklist is the easiest and understandable guidelines-based evaluation method. In the past research, many usability evaluation checklists are proposed but there does not exist a usability evaluation checklist that covers a maximum set of general as well as specific usability aspects for the evaluation of web-based library systems. To address this issue, the objective of this research is to create and propose a usability evaluation checklist for evaluating the usability of WBLS in Pakistan, where end user concerns encompass the complete functionality of WBLS, including both general and specific usability aspects. Following is a breakdown of the paper’s structure as “Literature review” section presented the literature review. The methodology is described in “Methodology” section.

The development of the proposed Usability evaluation checklist is discussed in “Development of usability evaluation checklist” section. The proposed checklist is evaluated in “Evaluation of proposed usability evaluation checklist” section. The results and analysis are discussed in “Results” section. Discussion about findings elaborated in “Discussion” section. “Threats to validity” section highlighted the threats to validity and Conclusion and future work are discussed in “Conclusion and future work” section.

## Literature review

Various research has identified the usability features of various WBLS. These checklists cover the majority of the generic features that apply to any WBLS and are used to assess any web-based user interface. Heuristic evaluation and evaluation of OPAC and NGC, on the other hand, found the most specific WBLS usability aspects.

In terms of general usability, studies covered aspects like the contact us link, user commenting or feedback and providing help to the user through online tutorials, search screen help, guide through documentation and FAQs that encourage the user to work with the site^12,14–25^. Many studies suggested the importance of the navigation aspect used to empowers the usability with the sub features including site map and table of content^12,15,16,19–24^. Many authors have addressed the need for informative, permanent and relevant links, return to a previous state or homepage link on every page addressed by many authors to boost the user pleasure^12,15–17,23,26–29^. The majority of studies emphasize the relevance of the search aspect of a website’s usability^12,15–17,23,24,28,30^. Many studies emphasize the importance of an attractive and well-organized homepage including images, color, organization’s logo, graphics and animation^12,15–17,19,29^. The appearance and pleasant user interface also an important aspects user satisfaction with the WBLS^26,27,31^. Various studies have emphasized the importance of data credibility that provides complete, relevant and updated information about the WBLS under the category about us link^12,15–17,19,23–25,29^. The usability of WBLS has been improved by several authors in terms of clear and understandable use of graphs, images, tables and diagrams with comprehensive viewpoint^12,15–17^. Many authors have raised concerns about inconsistencies in the site’s presentation of content, jargon, design, and navigation, all of which deter users. They shows that the user experience is influenced by the use of logical and consistent content, understandable text, and relevant and informative titles, labels, icons, and buttons^12,15,16,18,23,24,28,29,31^. Many studies have emphasized usability in terms of site responsiveness across all browsers, loading and downloading speed, and accessibility via simple and responsive URLs, as well as error prevention^12,15–17,23,24,28,29,31^. various factors of usability, such as presenting the site’s audience, offering membership to the user, and providing support via software tools such as PDF, visitor count, ad-free content, and other fun content, all of which were mentioned in the literature, contributed to better usability^12,15,21,22,25^. The importance of the copyright statement, bibliographical information of library content, separate display of new arrivals, and hyperlinks to external resources, e-journals, and open access resources has been highlighted in studies on the evaluation of specific usability aspects of library websites^17,18,21,22,25–27^. Various studies highlight the importance of specific WBLS aspects such as ask a librarian and reference assistance is rated higher including the RSS feed, instant messages and virtual help features of blogging and discussion forums^17,25–28^. The usability aspects addressed as simple keyword searches and advanced search helps in acquiring the required search results more quickly and accurately^18,20,26,27,32^. The specific usability aspects as facilitating the library sites with a spell checker, auto-correct and auto-complete natural language facility to search and safe search feature to prevent visibility of undesired content to children as provided by Google also enhances the user experience while using WBLS^20–22,26,27^. Studies also present sharing and exporting the searched content^18,20,32^, refine and modify to reset query at any stage of searching^18,19,28^, save the searched results and allow to download the library content^18^, sort and prioritize the results per date, subject or keyword raise the usability of the site^18,20^. Display of search results with a poor organization, irrelevant ordering, grouping and prioritizing the results negatively effects the usability of the site^19,20,26,27^. The studies also focus on the importance of recommending search results based on the number of hits on a library item or the content’s availability, which allows users to get speedy results on their preferred content^25,26^.

The literature analysis in Table 1 showed that the usability evaluation checklist for evaluating WBLS reveals most of the generic usability aspects. It is also analyzed that there does not exist an optimal set of specific usability aspects of WBLS in a single checklist. To identify and extract the specific usability aspects of WBLS the literature expanded to other usability methods and field such as OPAC, NGC and HEV that help to make the WBLS more successful and usable to the users in using domain specific features.

**Table 1:** Literature analysis: (where (√) shows that the particular aspect exists in the given literature (shows >  = 50 <  = 100% presence in literature), (×) represents that the particular aspect does not exist or 0%referred to by the particular literature and the symbol (≈) represents that the particular usability aspect is referred > 0 < 50% in the given literature).

Aspects	Usability checklists	HEV, OPAC and NGC
Interaction	(√ )	(≈)
Navigation	(√ )	(≈)
Aesthetic	(√ )	(≈)
Content	(√ )	(≈)
Content presentation	(√ )	(≈)
Consistency	(√ )	(≈)
Accessibility	(√ )	(×)
Error prevention	(√ )	(≈)
Miscellaneous	(√)	(≈)
Library content	(≈)	(√ )
E-sources	(≈)	(√ )
Library services	(≈)	(√ )
Smart searching	(×)	(√ )
Search results	(×)	(√)

Therefore, it is concluded from Table 1 that, there is not an optimal usability evaluation checklist proposed in the literature that helps the user to evaluate the general as well as specific usability aspects of WBLS. There is a lack of specific usability aspects such as smart searching and search results that affects the usability of WBLS in a negative manner. So, there is need to develop the effective and efficient usability evaluation checklist with maximum coverage of usability aspects that comprises the general as well as specific usability aspects to evaluate WBLS.

## Methodology

**Figure 1:**
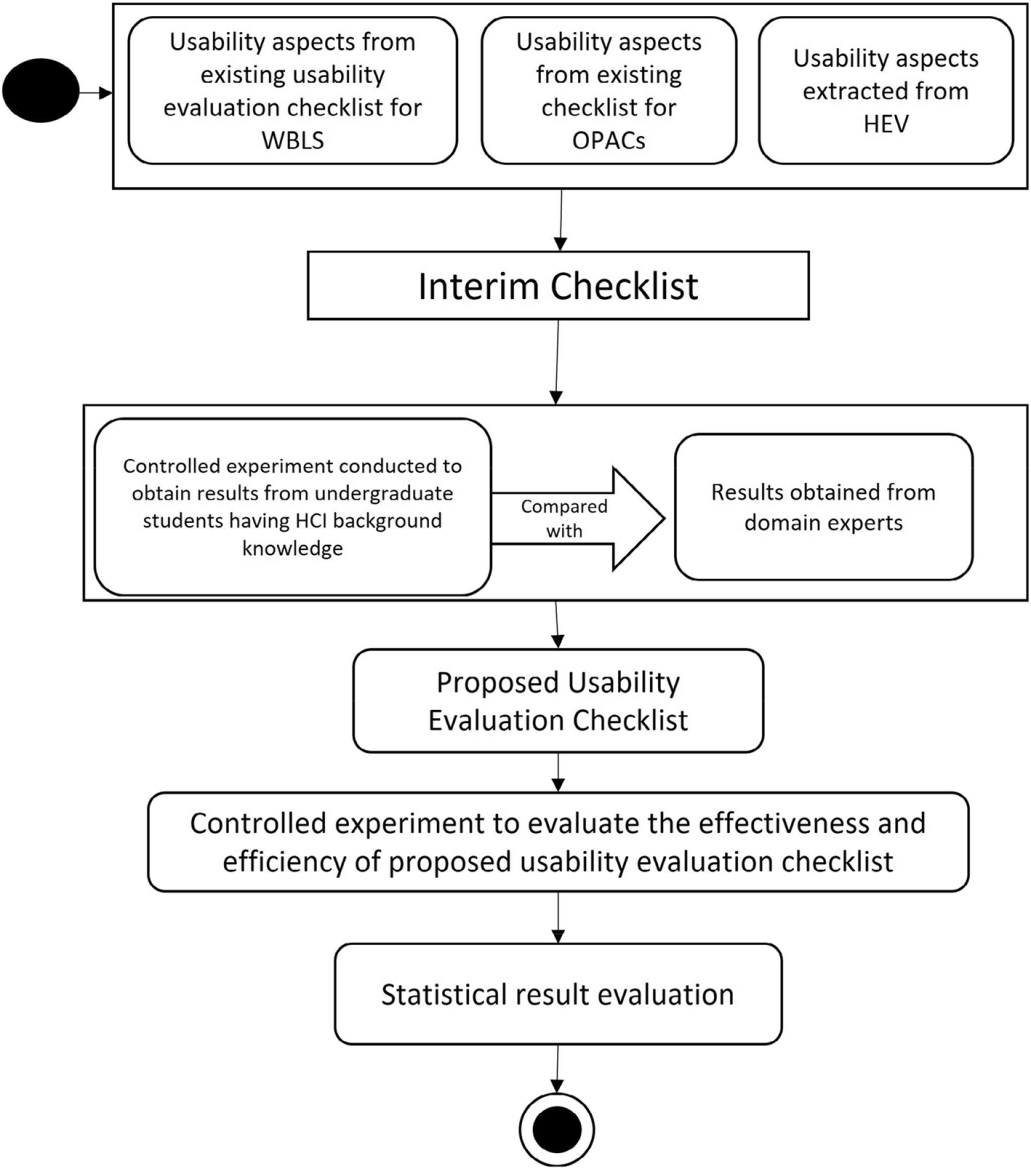
Proposed methodology.

The process in Fig. 1, presents an overview of proposed method for usability evaluation checklist. It is designed to address the limitations stated above in existing evaluation checklists for WBLS. The result of the process is to develop an effective and efficient usability evaluation checklist that maximizes the coverage of usability including general and specific aspects of WBLS.

The research questions of the study are:

RQ1: What is the level of coverage of usability aspects provided by different checklists in web-based library systems?
RQ2: How effective and efficient the proposed checklist is with respect to providing coverage of optimal set of usability aspects?

The questions are answered by following multiple steps described in detail below.

- In step 1, all the usability aspects from the existing checklists are elicited. Moreover, this study relied on elicitation of specific usability aspects from the checklist based and heuristic evaluation-based usability aspect from OPAC and NGCs. All the elicited usability aspects general and specific are integrated to form an interim checklist.
- In Step 2, first controlled experiment is conducted using interim checklist to evaluate 5 selected WBLS. The purpose of this step is to develop a checklist of optimal set of usability aspects general and specific that are required for evaluation of WBLS.
- In step 3, to eliminate the ambiguity and false results, the identified usability aspects are compared to the usability aspects identified by usability experts. In doing so, the usability aspects (general and specific) that really exists in WBLS are determined. These true usability aspects are used to create a proposed usability evaluation checklist.
- In steps 4, second controlled experiment is performed to evaluate the effectiveness and efficiency of proposed checklist in comparison to existing optimal checklist known as Academic Library Website Evaluation Checklist.
- In step 5, the results obtained are analyzed. The statistical t-test is applied to analyze the effectiveness and efficiency of proposed usability evaluation checklist.

## Development of usability evaluation checklist

This section explains how the usability evaluation checklist is developed. It explains how usability aspects are solicited and integrated from existing literature, and then how they are modified to create an optimal usability evaluation checklist, which is represented as an interim checklist.

### Interim checklist

The existing literature is reviewed to capture all the usability aspects to develop an interim checklist as shown in Table 2. Usability aspects general and specific to the WBLS domain are identified through existing usability evaluation checklist of WBLS, OPAC and NGC evaluation.

**Table 2:** Interim checklist.

Usability aspects	Sub-aspects	Usability questions
Interaction	Help	FAQ section available to answer user’s questions
		Online help provided in the site
		Site provides appropriate feedback about what is searched for
	Interactivity	Allow user to send feedback
		Allow user to comment on the site
		Contact us link provided on the site
Navigation	Navigation	Pages are easy to navigate (sitemap, path information, table of contents/index, navigation through scrolling, flipping and searching, back to home page)
		All provided links in site are working (no dead links)
		User can easily escape from undesired situation (back button, undo function, cancel/end program command)
	Shortcut	Shortcuts available in the site (bookmarks, table of content etc.)
	Searching	Internal search engine available to search library site
		Search result page is relevant to the searched term
		Allow user to search the library OPAC (Online Public Access Catalogue is online database of material held by the library)
	Links	Links are identifiable (through color or underline)
		Homepage link is on every page
		Links are closely associated with their reference
		Visited and unvisited links are clearly identifiable
Aesthetic	Interface design	Site is visually attractive
		Page layout is comprehensible
		Site is easy to use
	Graphics and animation	Easy to read graphics in the site (images, audio/video, relevant animation, flash technology, no watermarking)
		Graphics images are relevant to the context
		Site provides ALT tag for images (alternate text for an image, if the image cannot be displayed)
Content	Content accuracy	Site contains accurate information
		Pages are free of irrelevant information
		Most important features of the site easily recognizable
	Content currency	Site contains updated information
		Updated content satisfies the information need
		Shows the date/time the site was last updated
	Content completeness	Details of organization provided in the site
		Sufficient information presented to understand the particular focus
		Unrelated information is explained in the site
Content presentation	Text	Text have margin around
		Text is easy to read in contrast to their background
		Text has standard font size (12-point print type)
	Titling and labeling	Titles are suitable to portray the content
		Clearly defined areas are not labeled (search, login etc.)
		Technical terms are not used to the title label
	Language	Site language is user-friendly
	Presentation	Content presented in sequential order (based on date, title etc.)
		Information is presented in different formats (full text, abstract)
		Highest priority of content is emphasized
	Date and time	Date presented in international format (YYYY-MM-DD)
Consistency	Consistency	Pages layout is consistent
		Links are consistent with objective
		Information is consistent with mission statement
Accessibility	Accessibility	Loading/downloading speed is high
		Past content is accessible
Error prevention	Error prevention	Site allow user to recover from error (cancel operation, return to original)
		Site gives alert message to prevent from error
		Error prevention message reveals the description of the error
Misc	Ads	Site is free of ads
	Charges	Site provide free access to information resources (user don’t need to pay)
	Audience	Audience of the page stated (computer science, electrical engineering, BBA etc.)
	No of hits	Site shows the number of visitors in specified period of time
	Responsiveness	Site is responsive to (libraryname.com or www.libraryname.com)
	Tool assistance	Essential software is accessible from the site (like PDF etc.)
Library content	Library content	Site shows bibliographical details of main sources (books, research journals, authors etc.)
		Copyright of information resources stated
E-sources	E-sources	Site allow access to E-journals
		Access possible to open access resources
		Access possible to other web reference sites
Library services	Library services	Site allow user to ask from librarian
		Printing service available to user
		RSS feed is provided (Really Simple Syndication useful for keeping update on your favorite area through a feed like in blogs, newsletters, and podcasts)
		Site allow user to register as a member of the site
Smart searching	Smart searching	Site provide facility of advance search
		Facilitate the feature of auto correct/complete in searching
		Provide the facility of the spell checker in the site
		Provide an age appropriate “safe search” feature
		Site is able to handle “natural language” queries
		Site is able to retain queries from previous searches (search history)
Search results	Search results	Search result pages are relevant to the searched content
		Site response quickly in searching
		Allow user to export the records
		Site allow user to refine the search queries
		The site provides the faceted navigation. (Library catalogues should be able to display the search results as a set of categories, such as subject terms, dates, languages, availability, formats, locations, etc.)
		Site gives recommendation for related material
		Site provide facility of sharing the search records (social media interaction)
		Provides the facility of saving the search records
		Site gives recent search page results
		The site is able to prioritize the search results?

The interim checklist consists of eighty-two most identified usability questions; assembled into fourteen main aspects. General usability aspects that are most commonly found in literature are interaction, navigation, aesthetic, content, content presentation, consistency, accessibility and error prevention. However, Ads free sites, free access of information, audience, number of hits responsiveness and tool assistance like PDF, seldom addressed usability aspects that exist in literature. The domain specific usability aspects as library content, E-sources, library services, smart searching and search results are extracted from the checklist based and heuristic based evaluation of OPAC and NGC. Each usability aspects contain different relevant questions to collectively evaluate the particular aspect. The usability questions are formulated in such a way that firmly indicates and guides the explicit usability error in WBLS.

### Development of proposed usability evaluation checklist

The goal of development is to construct a usability evaluation checklist that satisfies the optimal and comprehensive set of usability aspects including general and specific to WBLS. A control experiment^33^ is performed to answer research question (RQ1). The participants used the interim checklist to evaluate the WBLS of five universities in Pakistan. A total of 86 subjects participated in the experiment. The participants are divided into group of five making a total of 430 subjects. Each group used the interim checklist and task scenarios on a different WBLS. The subjects of the experiment are chosen from students taking course of Human Computer Interaction, all being in the third year of bachelor’s in software engineering program. The participants are first given a training session of 30 min about the technique they are to use. The participants then use the technique on the assigned WBLS within an hour. The results on interim checklist obtained in the form of “Yes” and “No”, where “Yes” represent the existence of usability aspect and “No” represent the non-existence of usability aspects formally known as usability error. The evaluation criteria with fourteen usability aspects are interaction, navigation, aesthetics, content, content presentation, consistency, accessibility, error prevention, miscellaneous, library content, E-sources, library services, smart searching and smart results. The obtained results are measured by calculating the ratio between no of usability errors found per total no of usability errors that exist. The total number of usability errors are identified from expert of the field prior to execution of the experiment.

Table 3 illustrates that the interaction (71%), aesthetic (72%), consistency (79%), library content (78%) and E-sources (80%) are highly identified usability aspects in WBLS to enhance it usability. Whereas Navigation, content, content presentation, accessibility, Ads free sites, free access of information, audience, number of hits responsiveness and tool assistance like PDF are frequently identified. The usability aspect of error prevention and library services is rarely reported during evaluation.

**Table 3:** Identified level of coverage of usability aspects: (the identified true usability aspects ranked as high (> = 70), Medium (> = 50) and Low (< 50) classifications).

High usability aspects	Medium usability aspects	Low usability aspects
Interaction (71%)	Navigation (64%)	Error Prevention (49%)
Aesthetic (72%)	Content (68%)	Library Services (48%)
Consistency (79%)	Content Presentation (69%)	
Library Content (78%)	Accessibility (69%)	
E-Sources (80%)	Miscellaneous (66%)	
	Smart Searching (56%)	
	Search Results (54%)	

In the analysis of the specific usability aspects, the result shows that E-sources is ranked high during evaluation showing importance of access to E-journals, open access journals and web references to research libraries. Another domain specific aspect library content is also ranked high representing the importance of bibliographical details of book, journals, authors, and copyright features. The usability aspect as library services has least counts, which includes features as membership, online librarian assistance, printing facility, and RSS feed.

For development of the proposed usability evaluation checklist, each usability question under each aspect is ranked by defined high, medium, and low-ranking criteria. The proposed usability evaluation checklist represented in Table 4, consist of usability questions, which resulted as high (≤ 70) under each usability aspects.

**Table 4:** Proposed usability evaluation checklist.

Usability aspects	Sub-aspects	Usability questions
Interaction	Help	Online help is provided
		The site provides appropriate feedback about what is searched for
	Interactivity	Contact us link provided
Navigation	Navigation	Pages are easy to navigate (sitemap, path information, table of contents/index, navigation through scrolling, flipping and searching, back to homepage)
	Links	Links are identifiable (through colour or underline)
		Homepage link is on every page
		Links are closely associated with their reference
	Searching	Search result page is relevant to the searched term
		User Can search library OPAC (Online Public Access Catalogue is an online database of material held by the library)
Aesthetic	Graphics and animation	Easy to read graphics in the site (images, audio/video, relevant animation, flash technology, no watermarking)
		Graphics images are relevant to the context
	Interface design	The site is visually attractive
		Page layout is comprehensible
Content	Content accuracy	Information is accurate
		Important features of the site easily recognizable
	Content completeness	Details about the organization provided
		Sufficient information presented to understand the focus
	Content currency	The site contains updated information
		Updated content satisfies the information need
Content presentation	Content presentation	Content presented in sequential order (based on date, title etc.)
		Emphasize the highest priority of content
	Text	The text is easy to read in contrast to their background
		Text has standard font size (12-point print type)
	Titling and labelling	Titles are suitable to portray the content
	Language	Language is user-friendly
Consistency	Consistency	Pages layout is consistent
		Links are consistent with the objective
		Information is consistent with the mission statement
Accessibility	Accessibility	Past content is accessible
Miscellaneous	Ads	Free of ads
	Charges	Provide free access to information resources (user don’t need to pay)
Library content	Library content	Provide bibliographical details of main sources (books, research journals, authors etc.)
		Copyright of information resources stated
E-sources	E-sources	Access to E-journals
		Access possible to open access resources
		Access possible to other web reference sites
Library services	Library services	An option to ask from a librarian
Smart searching	Smart searching	The site provides the facility of advance search
Search results	Search results	Search result pages are relevant to the searched content
		Site response quickly in searching

The result shows that the identified level of coverage of usability aspects of a WBLS evaluation includes, interaction, navigation, aesthetic, content, content presentation, consistency, accessibility, ads, charges, library content, E-sources, library services, smart searching, and search results. Accordingly, the proposed usability evaluation checklist is comprehensive and optimal with maximum coverage of usability aspects (general and domain specific) under one checklist.

## Evaluation of proposed usability evaluation checklist

The goal of the evaluation is to assess the efficiency and effectiveness of the proposed checklist, in an academic context. A controlled experiment is conducted to answer research question (RQ2), which measures the general and domain specific usability aspects of two WBLS of universities of Pakistan.

The proposed checklist is compared with existing checklist known as Academic Library Website evaluation checklist^17^ which is most relevant to our context. The mentioned checklist is chosen because it contains the general and specific usability aspects to evaluate online libraries. A Latin-squares experiment is designed, dividing the participants into two groups; each group is given task scenarios and both checklists, but receives different treatment in term of WBLS. Twenty subjects participated in each session resulting in forty samples for each WBLS. It is sufficient number of subjects required to perform meaningful statistical analysis. The participants are given a training session of 30 min about the technique they have to use. The participants are then given 2.5 h to use the technique. T-test is applied on the results to ensure validity.

In this experiment, effectiveness and efficiency is measured. The effectiveness is calculated by the comparison of both evaluation checklists (proposed usability evaluation checklist and Academic Library Website Evaluation Checklist) with respect to the usability errors identified during evaluation. For this purpose, both checklists are compared to identify the common usability aspects, which are found as interaction, navigation, aesthetic, library content, E-sources, library services and search results.

Table 5 shows the common questions for effectiveness analysis under the matching usability aspects of both checklists. The results for effectiveness are collected using a quantitative rating system (ten-point scale), which signifies the level of existence of usability errors in particular WBLS. The rating scale (0–10) with rating (0–3) least existence, (4–6) for average existence and (7–10) for highest existence of usability aspects in WBLS, the usability errors are considered as usability aspects which have least existence in evaluation.

**Table 5:** Common usability aspects between both checklists for effectiveness analysis.

No	Proposed usability evaluation checklist	Academic library website evaluation checklist
1	Contact us link provided	Given all the contact information
2	Pages are easy to navigate (sitemap, path information, table of contents/index, navigation through scrolling, flip- ping and searching, back to home page)	Web pages are easy to navigate
3	Home page link is on every page	Every page included way to turn the home page for the site
4	Links are closely associated with their reference	All hyperlinks appropriate and relevant for an online reference desk
5	Easy to read graphics in the site (images, audio/video, relevant animation, flash technology, no watermarking)	The graphics and texts are most clear and easy to read
6	User Can search library OPAC (Online Public Access Catalogue is online database of material held by the library)	An option to search a library’s OPAC
7	Copy right of information resources stated	Copyright status are clearly stated
8	Access to E-journals	Hyperlinks to e-journals and databases
9	Access possible to other web reference sites	Hyper links to other web reference sites
10	An option to ask from librarian	An option to request reference assistance
11	Site response quickly in searching	Server appear to be fast

## Results

To measure the effectiveness of the proposed checklist, Table 6 summaries and compares the usability errors identified by both checklists, which shows a significant difference between both checklists. The major difference found in the aspects of interaction and library services where proposed checklist has identified 30 and 21 usability errors while only 6 and 7 usability aspects are identified using the Academic Library Website Evaluation Checklist. For the usability aspect, aesthetics, proposed checklist has identified 8 errors but in contrast existing checklist is unable to identify any error. In the aspect of navigation (14), E-sources (10), and search results (10) proposed checklist has identified maximum usability errors as compared to existing checklist (4). Both the checklists identify same usability errors for the aspect of library content (15). The cumulative results present that the proposed checklist has identified 108 usability errors, while existing checklist identified 49 usability errors. Therefore, the proposed checklist has more coverage of usability questions in each usability aspects as compared to the existing checklist. It also indicates that proposed checklist is more effective with maximum coverage of usability aspects i.e., interaction, navigation, aesthetics, library content, E-sources, library services, and search results.

**Table 6:** Effectiveness analysis of both checklists.

Usability aspect	Proposed usability evaluation checklist (check- list P)	Academic Library Website Evalu- ation Checklist (checklist E)
Interaction	30	6
Navigation	14	9
Aesthetic	8	0
Library Content	15	15
E-sources	10	8
Library services	21	7
Search results	10	4
Total	108	49

The independent sample t-test statistics is used to measure effectiveness and to determine the difference between two techniques. The results in Table 7 shows that, the significant difference between mean of two checklists is 0.033 which is less than the significance level of 0.05 that depicts the significance difference in the identification of usability errors. Using proposed checklist (M = 15.43, SD = 7.0) and Academic Library Website Evaluation Checklist (M = 7.74, SD = 4.62) conditions; t (12) = 2.47, p = 0.033. These results suggest that proposed checklist is an effective usability evaluation checklist. Specifically, the results suggest that proposed checklist results in identification of maximum usability errors.

**Table 7:** Statistical T-test results obtained for effectiveness analysis of both checklists.

	Checklist P	Checklist E
Mean	15.43	7.0
S.D (standard deviation)	7.74	4.62
T-test one tail	0.239	
T-test two tail	0.033	

To measure efficiency, the time taken by both checklists to evaluate the WBLS is compared. The result in Table 8 shows that proposed checklist took 27.8 min to find the usability errors, in contrast to Academic Library Website Evaluation Checklist, which consumed 27.6 min to find the usability errors. These results show that the rate of identifying usability errors per minute by both usability evaluation checklist is almost the same. Hence, it is concluded from the above discussion that the proposed checklist and the Academic Library Website Evaluation Checklist, both are efficient enough to identify the usability errors in equal time.

**Table 8:** Efficiency analysis of both checklists.

	Proposed usability evaluation checklist	Academic library website evaluation checklist
Average time in minutes	27.8	27.6

Table 9 shows the statistical t-test for comparison of efficiency of proposed checklist and Academic Library Website Evaluation Checklist applied, which shows significance difference as 0.965, which is greater than significance level 0.05. It means that there is no significance difference in the efficiency of proposed checklist (M = 0.690, SD = 0.465) and Academic Library Website Evaluation Checklist (M = 0.695, SD = 0.556) conditions; t (78) =  − 0.44, p = 0.965. The results suggest that both checklists P and E are equally efficient to identify the usability errors.

**Table 9:** Statistical T-test for efficiency of both checklists.

Height	Checklist P	Checklist E
Mean	0.690	0.695
S.D (standard deviation)	0.465	0.556
T-test one tail	0.404	
T-test two tail	0.965	

## Discussion

This section presents analysis on results and significance of research.

### Identifying general and specific usability aspects

This research uncovered several concerns related to the usability of WBLS for evaluation. Research indicated that the usability factors provided by existing studies fall short of covering an optimal set of usability features with general and specific usability aspects. Which varies depending on the checklists and heuristics used. However, general and specific usability aspects are extracted from selected studies and an interim checklist is produced. This aided in the processing of the study’s first research topic. (RQ1): “What is the level of coverage of usability aspects provided by different checklists in web-based library systems?” To answer RQ 1, The analysis of results obtained from the first experiment reveals that, the identified level of coverage of usability aspects for WBLS evaluation includes, interaction, navigation, aesthetic, content, content presentation, consistency, accessibility, ads, charges, library content, E-sources, library services, smart searching, and search results. Accordingly, the proposed usability evaluation checklist is comprehensive and optimal with maximum coverage of usability aspects including general and domain specific usability aspects under one checklist. However, the proposed checklist unable to identify the aspect of error prevention which is an important aspect in Nielson heuristics. One of the reasons can be that errors prevention is considered critical for data entry web-based applications, whereas WBLS mostly consist of search functionality. It could also be due to the study method, which does not contain such functionality in WBLS.

### Comparative analysis with existing checklists

(RQ2): “How effective and efficient the proposed checklist is with respect to providing coverage of optimal set of usability aspects?”.

The result analysis through the comparative method and statistical analysis shows that the proposed checklist is more effective than Academic Library Website Evaluation Checklist due to its maximum identification of general and specific usability errors. Since each usability, aspect in proposed checklist is covered via optimum numbers of usability questions than the Academic Library Website Evaluation Checklist. However, both the evaluation checklists are equivalent efficient. This may be because both checklists are easy to understand by the subjects, hence equal time is taken to perform a task. In comparison to the existing checklist, which exposed less usability errors in the same amount of time, the proposed approach is significant in detecting the most usability errors in less time. Furthermore, the existing checklist (checklist E) lacks in covering important usability aspects such as content completeness, content presentation, consistent layout and hyperlinks, bibliographical details of library-specific content such as books, journals, and most importantly, advance search and quick and relevant search results. The proposed checklist is significant for academia to evaluate their web-based library systems in term of its maximum coverage of general as well as specific usability aspects in an efficient manner with respect to time.

## Threats to validity

This section describes the various challenges we encountered during the research and how we attempted to tackle them. These are as follow:

### Internal validity

To reduce the risk of internal validity, the selected WBLS were selected based on open access and from Pakistani universities. To eliminate the bias of age and qualification level, the subjects are selected as undergraduate software engineering students with expertise of HCI. The interim checklist was developed in easy language based on the user perspective to lowers the possibility of learning effect and false WBLS evaluation. All subjects receive training to eliminate bias and understandability difficulties in the research procedure and domain. Furthermore, they are not subjected to any time constraints, allowing them to complete the inspection procedure with ease and without the risk of cognitive overload.

### External validity

To reduce the impact of external threats, the subjects were chosen from a number of Pakistani universities. To guarantee a balanced mix of individual capability, the sample is chosen independently and at random. The subjects are required to complete the experiment in a well-equipped computer lab, where access to resources such as computers and the internet ensures external validity. Participants were guaranteed marks in their final grades to keep their interest in the experiment alive.

### Conclusion validity

The reliability and understandability of the interim checklist/instrument are obligatory for avoiding conclusion validity; therefore, the pilot study conducted prior to experiment to remove the redundant questions and increased the worth of conclusion validity. Developing the usability evaluation checklist proved to be a challenging task for this study. There is a chance that the students giving false positive responses. To solve this issue, we did the same process with experts to collect the true usability aspects of the proposed checklist. Furthermore, the statistical t-test is used to compare the efficiency and effectiveness of both checklists and determine the statistical validate the results.

### Construct validity

To satisfy the construct validity, this research has developed the interim checklist by considering all usability checklist with the general usability aspects that are sifted with the questions of OPAC and NGC evaluation checklist and guidelines proposed by heuristic evaluation. Moreover, the usability experts carried out the validation of checklist.

## Conclusion and future work

Usability is an important quality attribute to be satisfied by the WBLS to its users. This research gave a deep understanding of a usability evaluation checklists checklist p and checklist E based on usability errors in web-based library systems and highlighting its general and specific usability aspects. Finally, a usability evaluation checklist has been evaluated by calculating its effectiveness and efficiency to evaluate web-based library systems. The proposed usability evaluation checklist is found to be effective in covering maximum usability aspects including general and specific to WBLS. and efficient enough to evaluate maximum usability in less time. The proposed usability evaluation checklist (checklist P) is important for academia to have a foundation for developing a quality library website for users based upon usability aspects and evaluating their existing web-based library systems to fulfill basic criteria. For future directions, the proposed usability evaluation checklist could be made more efficient by minimizing the time it takes to evaluate the WBLS. Another suggestion is to assess and improve the coverage of the proposed usability evaluation checklist by increasing the number of usability aspects. The proposed usability evaluation checklist can also be validated by creating a prototype of a web-based library system. This will guide the academia about the optimum standard of usability considerations for coverage of usability aspects.
